# Bismuth Quadruple Therapy with Doxycycline Is an Effective First-Line Therapy for *Helicobacter pylori* in an Irish Cohort

**DOI:** 10.3390/antibiotics14080757

**Published:** 2025-07-28

**Authors:** Conor Costigan, Mark Comerford, Ronan Whitmarsh, Kevin Van Der Merwe, Gillian Madders, Jim O’Connell, Thomas Butler, Stephen Molloy, Fintan O’Hara, Barbara Ryan, Niall Breslin, Sarah O’Donnell, Anthony O’Connor, Sinead Smith, Syafiq Ismail, Vikrant Parihar, Deirdre McNamara

**Affiliations:** 1 Trinity Academic Gastroenterology Group, Trinity College, D02 R590 Dublin, Ireland; thbutler@tcd.ie (T.B.); stmolloy@tcd.ie (S.M.); mcnamad@tcd.ie (D.M.); 2 Department of Gastroenterology, Tallaght University Hospital, D24 NR0A Dublin, Irelandniall.breslin@tuh.ie (N.B.); sarah.odonnell@tuh.ie (S.O.); anthony.oconnor@tuh.ie (A.O.); simthsi@tcd.ie (S.S.); 3 Department of Gastroenterology, Cavan Monaghan Hospital, H12 VK68 Cavan-Monaghan, Ireland; ronan.whitmarsh@hse.ie (R.W.); syafiq2009@yahoo.com (S.I.); 4 Department of Gastroenterology, Letterkenny University Hospital, F92 AE81 Donegal, Ireland; kvandm91@gmail.com (K.V.D.M.); vikpar37@yahoo.coml (V.P.)

**Keywords:** *Helicobacter pylori*, *H. pylori*, *H. pylori* eradication, bismuth quadruple therapy

## Abstract

**Background:** There has been a reduction in successful *H. pylori* eradication rates recently, which is largely attributed to increasing antibiotic resistance. In areas of high dual clarithromycin and metronidazole resistance such as ours, Maastricht VI/Florence guidelines recommend bismuth quadruple therapy (BQT) as first line of therapy; however, the availability of bismuth was poor in Ireland until recently. Similarly, tetracycline, a component of BQT, is restricted locally, with doxycycline (D) being approved and reimbursed for most indications. Aims: To assess the efficacy of BQT-D therapy for *H. pylori* eradication in an Irish cohort. **Methods:** All patients testing positive for *H. pylori* in three Irish referral centres by urea breath test, stool antigen, or histology were treated prospectively with BQT-D (bismuth subcitrate 120 mg QDS, metronidazole 400 mg TDS, doxycycline 100 mg BD and esomeprazole 40 mg BD) for 14 days. Eradication was evaluated with a urea breath test (UBT) >4 weeks after therapy cessation or by stool antigen testing, as available. Outcomes were recorded and analysed according to demographics and *H. pylori* treatment history of the patients. **Results:** 217 patients completed post-eradication testing. Of which, 124 (57%) were female, with a mean age 52 years. 180 patients (83%) were treatment-naïve. A total of 165/180 (92%) of the treatment-naïve patients had successful eradication. There was no association between eradication and gender or age in this cohort (*p* = 0.3091, *p* = 0.962 respectively). A total of 29 patients received this therapy as second-line therapy, of which 22 (76%) had successful eradication. Eight patients received the regimen as rescue therapy, with seven (88%) having successful eradication. No serious adverse events were reported. Eleven individuals (6.5%) commented on the complicated nature of the regimen, with 11 tablets being taken at five intervals daily. **Conclusions:** BQT-D as first-line therapy for *H. pylori* infection is highly effective in a high dual-resistance population, achieving >90% eradication. BQT-D as a second-line treatment performed less well. Our data support BQT-D as a first-line treatment.

## 1. Introduction

*Helicobacter pylori* (*H. pylori*) is a bacterium which colonises and infects the human gastric epithelium, causing chronic gastritis, peptic and duodenal ulceration, mucosal associated lymphoid tissue (MALT) lymphoma, and gastric adenocarcinoma [1,2]. In 1994, it was classified by the World Health Organisation as a class-1 carcinogen [3] and, in 2015, the Kyoto Global Consensus Report on *H. pylori* classified it as an infectious disease, mandating eradication therapy in all diagnosed cases [4].

Although antimicrobial guidelines for *H. pylori* vary worldwide based on local antimicrobial sensitivities, bacterial culture and antimicrobial resistance testing for *H. pylori* can be technically difficult, while genetic testing for resistance genes is expensive and does not always show 100% concordance with clinical resistance patterns. Local drug availability is also a factor that affects the choice of therapy. Traditionally, European guidelines have relied on ‘triple therapy’ consisting of amoxicillin, a proton pump inhibitor (PPI), and either clarithromycin (CTT) or metronidazole (MTT) for eradication. Increased community resistance to these agents has corresponded to a decrease in the successful eradication rates in Ireland and many European countries [5,6]. In the face of ever-evolving resistance patterns, it is imperative that we investigate new antimicrobial regimens to achieve the >90% eradication target which is recommended [7,8,9]. Ireland is currently classified as an area of high dual resistance, that is primary resistance to both clarithromycin and metronidazole resistance is >15% [6,10,11].

In 2022, the European Helicobacter and Microbiota Study Group published the Maastricht VI/Florence consensus report on the treatment of *H. pylori* at a European level and have recommended that, in areas of high dual clarithromycin and metronidazole resistance, empiric treatment with bismuth quadruple therapy (BQT) should be considered as the first-line treatment, with an option for high-dose amoxicillin dual therapy (HDADT), if validated locally [8]. Unfortunately, a recent study from our group showed that HDADT is unlikely to be a candidate therapy in an Irish population due to poor eradication rates [12]. Similarly, the Maastricht VI guidelines recommend levofloxacin quadruple therapy as the second-line treatment; however, concerns regarding the potential adverse events associated with fluoroquinolone use and national warnings restricting their use have also limited the uptake of Levofloxacin-based regimens in Ireland [13,14].

Until recently, bismuth-based medications were not widely available, while combination tablets also remain unavailable nationally, and, as such, the adoption of bismuth-based quadruple therapy among clinicians had been slow and data validating its efficacy locally is lacking. Recently, the Irish guidelines on the management of *H. pylori* infection were updated to recommend BQT consisting of tetracycline 500 mg four times daily (QDS), metronidazole 400 mg three times daily (TDS), esomeprazole twice daily (BD), and bismuth subcitrate 120 mg QDS as first-line therapy, with a caveat that, although possibly less effective, based on limited international data [15,16], doxycycline may be substituted for tetracycline for medical or economic reasons [17].

Currently, in Ireland, doxycycline is approved for use and full reimbursement under the Health Service Executive’s Drugs Payment scheme [18]. This means that patients with a relevant indication can receive this medication for minimal or no cost to themselves. Tetracycline, on the other hand, is not reimbursable, and is expensive. Both tetracycline and doxycycline are part of the tetracycline family of antimicrobials. Tetracycline is naturally occurring, while doxycycline is semi-synthetic. They are both bacteriostatic in nature, acting as protein synthesis inhibitors on the 30S ribosome, and have wide antibacterial, antimalarial, anthelmintic, and anti-inflammatory effects [19,20].

This class of drugs has a common side effect profile that includes diarrhoea, nausea, and abdominal pain. Cautions are given to patients regarding increased photosensitivity, possible alterations to the metabolism of the oral contraceptive pills and anticoagulants, the risk of teratogenicity in pregnancy, and the possible effect of demineralising and staining of enamel and bone in children and adolescents [21,22]. However, some pharmacodynamic differences do exist between doxycycline and tetracycline.

Rash, headache, and dizziness are common features that are specific to tetracycline, while the longer half-life of doxycycline allows for a reduced dosing frequency [23]. Doxycycline demonstrates 100% gastrointestinal absorption compared with a maximum of 88% for tetracycline and, although both rates are adversely affected by meal consumption, doxycycline is less so (50% vs. 20%, respectively) [24,25,26]. Excretion of these drugs is primarily by the renal route; however, doxycycline may also be excreted in faeces, via the biliary route, which reduces the possibility of toxicity in patients with renal failure [27,28,29].

In this study, we aimed to assess the efficacy of BQT with doxycycline (BQT-D) therapy for *H. pylori* eradication in an Irish cohort.

## 2. Results

**Figure 1:**
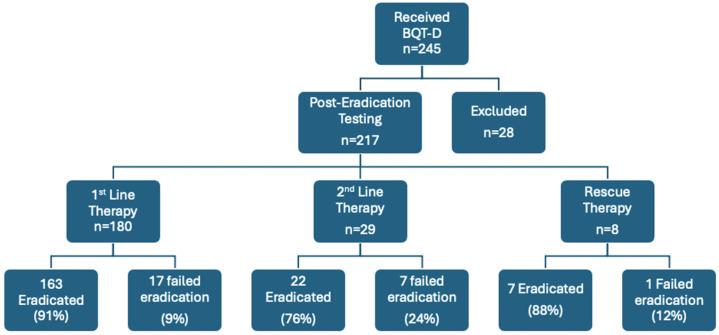
Results for BQT-D—bismuth quadruple therapy with doxycycline.

In all, 245 patients were treated prospectively with BQT-D during this study’s timeframe (Figure 1).

A total of 28 patients were excluded as a result of post-eradication testing not being completed within the timeframe of the study due to interval antibiotic use or continuation of PPI medication.

A total of 217 (89%) patients were included in the final analysis (n = 105, 86, and 26 for TUH, CMH, and LUH, respectively); the mean age was 52 ± 24.5 years and 124 participants (57%) were female (Table 1). The populations recruited in the three centres were statistically similar. In all, 180 (83%) patients received BQT-D as a first-line therapy, 29 (13%) as second-line therapy, and 8 (3.5%) as rescue therapy (≥2 previous therapies) (Table 1).

The overall eradication rate for the entire cohort, regardless of *H. pylori* treatment history, was 91%. Univariate analysis revealed that the overall eradication rates across the three hospital sites were similar (87%, 93%, and 88%, for TUH, CMH, and LUH, respectively, *p* = 0.686). Neither gender nor age had a significant effect on the eradication rates (*p* = 0.669, and *p* = 0.303 respectively).

A total of 165 patients out of the 180 (92%) in the first-line group had successful eradication, and the first-line eradication rates were similar across the hospital sites (66/72 or 92%, 78/84 or 93%, and 21/24 or 88%, for TUH, CMH, and LUH, respectively *p* = 0.833). Univariate analysis showed that the first-line eradication rates were similar between males and females (72/82 = 88% vs. 91/98 = 93%, respectively *p* = 0.962) and logistic regression determined that the same was true across ages (*p* = 0.962). The second-line and rescue therapy eradication rates were 76% (22/29) and 88% (7/8), respectively (Table 2). The difference in eradication rates between the naive and prior treatment groups was statistically significant (92% (165/180) vs. 78% (29/37), respectively, *p* = 0.0002).

A total of 28 of the 29 second-line patients had previously received CTT. The eradication rate for this group, post BQT-D, was 23/28 (82%). The remaining second-line patient had been treated with high dose amoxicillin dual therapy initially, and BQT-D treatment was unsuccessful.

Among the patients who received rescue therapy, the eradication rate was 7/8 (88%); however, a multitude of regimen types and a number of antibiotic courses had previously been administered, which prevented further meaningful statistical analysis of this group.

The self-reported compliance with >90% of the antimicrobial regimen was 100% at the time of post-eradication testing. There were no major adverse drug reactions (ADRs) reported. A total of 105 patients (48%) partook in a post-study phone interview, of which 9 (8.5%) reported minor GI upset that did not impact the completion of the antibiotic course and 11 (10.5%) commented on the complicated nature of the medication administration, with 11 individual tablets needing to be taken at five different times throughout the day.

## 3. Discussion

Our prospective real-world study across three Irish sites, with high dual clarithromycin and metronidazole resistance, validates the use of BQT-D as a first-line therapy in our cohort. First-line BQT-D reached the international 90% successful eradication target [8], was similar across sites, and was not affected by age or gender. Recent Irish and European guidelines have recommended BQT with tetracycline (BQT-T) as first-line therapy, but evidence for its superiority over doxycycline, in particular in our population, is scant.

Data extracted retrospectively from the European Registry on *Helicobacter pylori* management (Hp-EuReg) favoured tetracycline-based BQT; however, this study dealt only with Spanish patients undergoing third-line therapy and the number of patients who underwent 14-day therapy was small (n = 50 in each group) [30]. In addition, the PPI doses and prior treatments varied.

One large retrospective study from the US did show a difference in 14-day BQT-T compared to BQT-D (85% vs. 67%, *p*  =  0.006); however, neither reached the 90% eradication threshold that is required to be recommended [17]. Another retrospective analysis, also from the US, showed equal eradication rates of 88% for both regimens [31].

A meta-analysis of six case-controlled studies found no difference in efficacy (OR= 0.95; 95%CI 0.68−1.32; *p =* 0.77) [32]. Unfortunately, only two of these studies directly compared bismuth- vs. tetracycline-based bismuth regimens, with one favouring tetracycline and the other showing no difference.

BQT-D has been shown to be effective in a meta-analysis from China that was published in 2021 [33]. Data for this first-line therapy in European populations, however, are lacking. Our study represents the largest prospective study in a European patient population that attempts to validate this regimen.

Doxycycline use over tetracycline may confer specific advantages to patients.

In Ireland, doxycycline is fully covered under the government’s public health service drugs payment scheme, with an out-of-pocket saving for each patient. This reflects the reality of prescribing in the Irish public health system, as many patients have difficulty accessing expensive medications in the current economic climate.

The twice-daily dosing of doxycycline over four-times-daily dosing of tetracycline also simplifies an already complex dosing regimen, which is likely to improve patient satisfaction and therefore compliance. This is reflected by the large number of patients (10.5%) who volunteered the regimen’s complexity as a logistical issue in their post-study phone interview. In fact, further data from the Hp-EuReg suggest that the simpler dosing regimen associated with single-capsule therapy increases eradication rates when compared with therapy of the same constituent medications administered separately [34]. Further studies should continue to be conducted when the availability of single-capsule regimens is expanded to include Ireland.

Historically, BQT was reserved for second-line or rescue therapy in Ireland. While the numbers are small (n = 29) in our cohort, the BQT eradication rates in this setting were not adequate at 78%, and were similar for second-line or rescue therapy. Further investigation is warranted into more effective second-line and rescue therapies.

There are several drawbacks with this study. Not all patients returned for follow-up in the allotted time frame. Some required prolonged PPI therapy to ensure ulcer healing, while others were prescribed subsequent courses of antibiotics for other indications and were thus ineligible for post-eradication testing. Some patients were unable to attend post-eradication appointments for personal reasons and rescheduled appointments outside of the data collection window. However, this does reflect the reality of following up on outpatient testing in this population.

It is important to note that this is not a direct head-to-head trial between BQT with doxycycline and that with tetracycline. While we cannot comment on the superiority of one drug regimen over the other, we can say that BQT-D reached the 90% eradication threshold.

As these three centres are early adopters of BQT in Ireland, and as the participants in this study were taking part in a pilot study, it is likely they were given more attention by healthcare professionals, being closely followed up with by hospital staff and receiving more enhanced patient education than might occur in the general outpatient or community healthcare setting. It is likely that this positivity impacted both compliance with therapy and willingness to engage with follow-up testing.

This study suggests that BQT with doxycycline is a viable first-line treatment of *H. pylori* in an Irish cohort. In contrast to traditional BQT with tetracycline, it is cheaper, monetarily reimbursable by the health service to the patient at the point of access, and has the potential to increase the availability of this treatment for those for whom tetracycline may be contraindicated. BQT should no longer be reserved for second-line or rescue therapy.

## 4. Methods

### 4.1. Patients

This was a prospective open-label study performed across three tertiary referral centres in Ireland: Tallaght University Hospital (TUH), Cavan-Monaghan Hospital (CMH), and Letterkenny University Hospital (LUH). After approval by the ethics committee in our institution, which was undertaken as a quality improvement initiative, and similar authorisation in the other centres, patient recruitment began locally. Unselected adult patients > 18 years of age diagnosed with *H. pylori* via the urea breath test (UBT) with 50 mg urea (a delta-over-baseline >4% was considered positive), stool antigen testing or upper endoscopy (histology ± rapid urease test positive) were sequentially enrolled. All patients underwent the above testing for investigation of dyspepsia or reflux-like symptoms at their local hospital and were recruited into the study based on positive results. Exclusion criteria included known or suspected antibiotic allergy, refusal to consent to or to attend post-eradication testing within the allotted time frame or continuation of PPI or antibiotic therapy when post-eradication testing was due.

### 4.2. Regimen

All patients were prescribed a 14-day course of BQT-D: bismuth subcitrate (120 mg QDS), esomeprazole (40 mg BD), metronidazole (400 mg TDS), and doxycycline (100 mg BD). At least 4 weeks after the cessation of antimicrobial therapy, a post-eradication ^13^C UBT was performed using our standard protocol with 50 mg urea (Diabact, Laboratoires Mayoly Spindler, Chatou, France) or stool antigen testing (IMMUNOCARD STAT!^®^ HPSA^®^, Meridian Bioscience, Cincinnati, OH, USA), as available to the local hospital, to evaluate post-eradication *H. pylori* status. All patients were advised to avoid PPI for 7 days and antibiotics for 28 days before attending their post-eradication test. A delta-over-baseline >4% was considered positive for post-eradication UBT and used to confirm eradication in all patients from TUH. Detectable levels of *H. pylori* antigen (>64 ng/mL) in the stool were considered positive for stool antigen testing, and were used to confirm eradication in all patients attending both CMH and LUH. At the end of this study, compliance with therapy and side effects were assessed by phone interview. Self-reported administration of ≥90% of the medication course was considered compliant.

### 4.3. Data Analysis and Statistics:

Patient demographics (age, sex) and *H. pylori* treatment histories were recorded locally by the physician-scientists. *H. pylori* testing data were accessed from the GI lab, UBT database, or endoscopy database as appropriate in each hospital. The data were anonymised locally, collated centrally on a secure database with the data from the other hospital sites, and analysed with appropriate statistical methods. The effects of the demographic data on the eradication rates were analysed via the Fisher Exact test, logistical regression, or the Chi squared test where appropriate. In all cases, a *p* < 0.05 was considered significant. While the overall number of participants who received BQT-D are reported upon in this study, only those patients who retuned for post-eradication testing were included in the final analysis.

## 5. Conclusions 

BQT-D as first-line therapy for *H. pylori* infection is highly effective in a high dual-resistance population, achieving >90% eradication. BQT-D as a second-line treatment performed less well. Our data support BQT-D as a first-line treatment.

## Figures and Tables

**Table 1:** Study population.

	TUH	CMH	LUH	Total
N =	105	86	26	217
Female	64 (61%)	47 (54.5%)	13 (50%)	124 (57%)
Mean age (yrs)	50 ± 23.5	52 ± 25	61 ± 20	52 ± 24.5
1st Line (%)	72 (69%)	84 (98%)	24 (92%)	180 (83%)
1st line Age	50 ± 26	52 ± 23	61 ± 20	53 ± 25
2nd Line	25 (24%)	2 (2%)	2 (8%)	29 (13%)
Rescue Therapy	8 (8%)	0	0	8 (3.5%)

TUH: Tallaght University Hospital; CMH: Cavan-Monaghan Hospital; LUH: Letterkenny University Hospital.

**Table 2:** Eradication rates.

	TUH	CMH	LUH	Total
Total N =	105	86	26	217
Overall Eradication (%)	91 (87%)	80 (93%)	23 (88%)	194 (89%)
1st Line Eradication (%)	66/72 (92%)	78/84 (93%)	21/24 (88%)	165/180 (92%)
2nd Line Eradication (%)	18/25 (72%)	2/2 (100%)	2/2 (100%)	22/29 (76%)
Rescue Therapy Eradication (%)	7/8 (88%)	-	-	7/8 (88%)

TUH: Tallaght University Hospital; CMH: Cavan-Monaghan Hospital; LUH: Letterkenny University Hospital.

## Data Availability

The data presented in this study are available on request from the corresponding author due to Irish and European legislation regarding the sharing of personal and/or health information.
